# Post-weaning Social Isolation in Male and Female Prairie Voles: Impacts on Central and Peripheral Immune System

**DOI:** 10.3389/fnbeh.2021.802569

**Published:** 2022-01-17

**Authors:** Meghan L. Donovan, Eileen K. Chun, Yan Liu, Zuoxin Wang

**Affiliations:** ^1^Program in Neuroscience, Department of Psychology, Florida State University, Tallahassee, FL, United States; ^2^Rocky Mountain Mental Illness Research Education and Clinical Center, Rocky Mountain Regional VA Medical Center, Aurora, CO, United States; ^3^Department of Physical Medicine and Rehabilitation, University of Colorado Anschutz Medical Campus, Aurora, CO, United States

**Keywords:** social isolation, inflammation, cytokine, microglia, anxiety

## Abstract

The socially monogamous prairie vole (*Microtus ochrogaster*) offers a unique opportunity to examine the impacts of adolescent social isolation on the brain, immune system, and behavior. In the current study, male and female prairie voles were randomly assigned to be housed alone or with a same-sex cagemate after weaning (i.e., on postnatal day 21–22) for a 6-week period. Thereafter, subjects were tested for anxiety-like and depressive-like behaviors using the elevated plus maze (EPM) and Forced Swim Test (FST), respectively. Blood was collected to measure peripheral cytokine levels, and brain tissue was processed for microglial density in various brain regions, including the Nucleus Accumbens (NAcc), Medial Amygdala (MeA), Central Amygdala (CeA), Bed Nucleus of the Stria Terminalis (BNST), and Paraventricular Nucleus of the Hypothalamus (PVN). Sex differences were found in EPM and FST behaviors, where male voles had significantly lower total arm entries in the EPM as well as lower latency to immobility in the FST compared to females. A sex by treatment effect was found in peripheral IL-1β levels, where isolated males had a lower level of IL-1β compared to cohoused females. Post-weaning social isolation also altered microglial density in a brain region-specific manner. Isolated voles had higher microglial density in the NAcc, MeA, and CeA, but lower microglial density in the dorsal BNST. Cohoused male voles also had higher microglial density in the PVN compared to cohoused females. Taken together, these data suggest that post-weaning social housing environments can alter peripheral and central immune systems in prairie voles, highlighting a potential role for the immune system in shaping isolation-induced alterations to the brain and behavior.

## Introduction

Adolescence is a critical time window of development, where one’s environment can have long-lasting impacts on health outcomes. During this adolescent window, humans rely on a wide variety of social relationships to develop skills necessary for becoming successful adults. These adolescent bonds, such as with peers and family, provide necessary support for optimal wellbeing throughout life (Viner et al., 2012; Wang R.A.H. et al., 2017). In contrast, a lack of social support, or social isolation, during adolescence can be associated with profoundly negative effects on mental health, and can correlate with increased rates of both anxiety and depression in adulthood (Butler et al., 2016; Cruz et al., 2016; Matthews et al., 2016; Tzanoulinou and Sandi, 2016; Walker et al., 2019). Further, the perception of social rejection and social isolation is often correlated with dysregulation of immune and stress response systems in the body (Slavich et al., 2010; Hawkley et al., 2012; Murphy et al., 2013). Studies in humans have found that increased social isolation and perceived loneliness are also associated with elevated inflammation (Eisenberger et al., 2017; Leschak and Eisenberger, 2019). These long-term changes to physiological systems can have severe impacts on health and mortality. However, the specific underlying mechanisms of how isolation during adolescence can lead to detrimental behaviors and immune dysregulation remains largely unknown.

Traditional laboratory rodents have been used to better understand how adolescent social isolation alters behavior, the brain, and the immune system. In both mice and rats, social isolation immediately after weaning (a time window representative of adolescence, prior to adulthood) can result in increased anxiety-like and depressive-like behaviors (Lukkes et al., 2009a; Amiri et al., 2015; Huang et al., 2017; Walker et al., 2019). Social isolation not only increases maladaptive behaviors, but also changes the underlying neurocircuitry of the brain. For example, social isolation stress alters several neurochemical systems, including oxytocin (OT), and dopamine (DA), in brain regions involved in stress responses, such as the paraventricular nucleus of the hypothalamus (PVN) and dentate gyrus of the hippocampus (DG) (Tanaka et al., 2010; Oliveira et al., 2019). Furthermore, traditional animal models have been useful for understanding specific central and peripheral immune alterations that can result from developmental social isolation. For example, microglia, the primary immune cells of the brain, play a vital role in homeostatic regulation of the brain and are critical in synaptic pruning in early life for proper development of social play in male rats (Bilbo and Schwarz, 2009). Early life social stressors can also increase both microglial activation and cytokine release in the hippocampus in both mice and rats (Delpech et al., 2016; Dunphy-Doherty et al., 2018). Further, these microglial alterations have been shown to play a causal role in the resulting alterations in neuronal signaling and ultimate increases in depressive-like behaviors from isolation in rats (Wang H. T. et al., 2017).

Other social isolation studies have shown long-term dysregulation in both central and peripheral immune function as well (Chida et al., 2005; Ko and Liu, 2015). Interestingly, manipulation of the human immune system via peripheral inflammatory challenge increases perceived social disconnection (Eisenberger et al., 2010). Likewise, individual housing in adult rats elevated the levels of peripheral cytokines and increased display of “despair” behavior (Krügel et al., 2014). Peripheral cytokines have also been found to be essential for driving social behavior in traditional rodent models (Hoogenraad and Riol-Blanco, 2020; Reed et al., 2020). Taken together, these studies suggest a critical role of both the peripheral and central immune systems in shaping outcomes from isolation stressors. Although these studies lay out a strong foundation for uncovering mechanisms of isolation, most studies to date have been done in species that do not exhibit some of the social behaviors humans show (i.e., forming social preferences, biparental care, etc.). To fully understand how isolation during developmental windows can alter central and peripheral immune function and behaviors, a highly social animal model that displays these translational behaviors must be used.

The prairie vole (*Microtus ochrogaster*) is an established rodent model that displays a myriad of social behaviors. As a socially monogamous species, the prairie vole has been used to investigate the neurobiological mechanisms underlying the regulation of social monogamy, and several neurochemical systems, including DA and OT, have been implicated in regulating these social behaviors (Young et al., 2011; Lieberwirth and Wang, 2014; Tabbaa et al., 2017). Similar to humans, social isolation in prairie voles can be a powerful stressor. Data from previous studies have shown that isolation in adult prairie voles alters the gut-immune-brain axis (Donovan et al., 2020), increases depressive-like behaviors (Grippo et al., 2008), and alters both plasma OT and central OT expression in the PVN (Grippo et al., 2007a). Interestingly, adult prairie voles housed in isolation show alterations in both immune function in the peripheral nervous system (Scotti et al., 2015) and autonomic function (McNeal et al., 2014), supporting the notion that post-weaning isolation may have system-wide effects in the periphery as well. However, it is still unknown how social isolation during development can affect central and/or peripheral immune function and how these alterations may differ across sexes.

As prairie voles often live in the nest through adulthood in the wild (Getz et al., 1993; Getz and Carter, 1996), the lack of social environment during adolescence (i.e., post-weaning, but prior to reaching adulthood) can be detrimental. For example, a previous study showed that male prairie voles exposed to 6 weeks of isolation after weaning display increased anxiety-like behaviors and altered mRNA levels for OT, vasopressin (AVP), corticotrophin releasing hormone (CRH), and tyrosine hydroxylase (TH) in the PVN (Pan et al., 2009), suggesting that post-weaning isolation can alter a wide variety of neurochemical systems in prairie voles. This 6-week period after weaning can be representative of a juvenile, developmental window for prairie voles, as they reach adulthood/reproductive maturation around postnatal day 60–80. Moreover, the manipulation of social factors (i.e., presence of siblings, population density, etc.) has been shown to alter juvenile vole behaviors (Getz and Carter, 1980, 1996; McGuire et al., 1993), emphasizing the importance of social cues during this time frame. Therefore, in the present study, we examined how social isolation via single housing during a developmental period that begins at PND 21–22 (i.e., post-weaning) and lasts until prairie voles reach adulthood (after PND 60) may differentially alter microgliosis in the brain and circulating levels of cytokines in both male and female prairie voles. We hypothesized that post-weaning social isolation has enduring effects on behaviors as well as on peripheral and central immune marker expression, and such effects may differ between male and female prairie voles.

## Materials and Methods

### Subjects and Social Isolation Procedure

Subjects were male and female prairie voles (*Microtus ochrogaster*) captive-bred at Florida State University and were kept at 20°C under a 14:10 h light:dark cycle (lights on at 0700). At weaning (PND 21-22), subjects were placed into a clean, Plexiglas cage (20 × 25 × 45 cm) with a standard metal cage lid and were randomly assigned to either be housed alone (social isolation) or with a same-sex cagemate (a standard housing condition for prairie voles laboratory settings) (*n* = 10 per sex per treatment; *n* = 40 total), using our previously established methods (Pan et al., 2009). All cages contained cedar chip bedding with food (Purina 5326 pelleted rabbit food, large pellet form) and water provided *ad libitum* by Laboratory Animal Resources staff. Isolated subjects were in the same room as cohoused subjects but were placed on separate rows of housing racks from cohoused animals. Although isolated animals were singly housed to prevent social contact throughout the treatment period, they could still possibly receive olfactory/auditory/visual cues from the other animals in the housing room. The isolation/cohousing procedure lasted 6 weeks, after which all subjects underwent behavioral testing. All procedures were approved by the Institutional Animal Care and Use Committee at Florida State University and were in accordance with the guidelines set forth by the National Institute of Health.

### Behavioral Testing

All subjects underwent the elevated plus maze (EPM) on day one of behavioral testing. The EPM has been established and validated in our previous vole studies (Pan et al., 2009; Smith and Wang, 2014). Briefly, the apparatus is elevated 45 cm off the ground and consists of two open (35 × 6.5 cm) and two closed arms [35 × 5 × 15 (H) cm] that cross in the middle. All subjects underwent EPM testing in the morning (between the hours of 0800 and 1000) on day one of behavioral testing. Subjects were placed onto the center of the maze facing an open arm, and their behaviors on the EPM were recorded for 5 min using Active Webcam software and were subsequently quantified by an observer blind to treatment via J-Watcher. Behaviors quantified included the duration and frequency in the open arms, closed arms, and the center of the maze. Locomotor activity (as indicated by total arm entries) was also calculated. Immediately after the EPM test, subjects were returned to their respective housing cages/conditions.

All subjects underwent the forced swim test (FST) in the morning (between the hours of 0800 and 1000) on day two of behavioral testing. The FST has been established and validated in our previous studies (Lieberwirth et al., 2012, 2013). Briefly, a large, Plexiglas tub [25 × 45 × 20 (H) cm] is filled with lukewarm water (32 ± 1°C). Subjects were placed into the tub and allowed to swim freely for 5 min. Subjects’ behaviors were recorded for the entire test using Active Webcam software and were subsequently quantified by an observer blind to treatment via J-Watcher. Behaviors quantified included the duration and frequency of swimming and immobility. Total activity bouts (locomotor measure) and the latency to immobility were also calculated.

### Blood Sample Preparation and Plasma Cytokine Analysis

After the FST, subjects were deeply anesthetized via euthasol and cardiac blood samples were collected immediately prior to perfusion. Blood was collected in microcentrifuge vials with 20 μl EDTA and were placed immediately on ice. Samples were then centrifuged at 6,000 rpm for 15 min at 4°C, and plasma was then transferred to new tubes. Plasma was re-centrifuged at 6,000 rpm for 10 min at 4°C. Plasma samples were then analyzed for cytokines on a 96-well plate in duplicates using the Milliplex MAP Cytokine magnetic bead panel (#MCYTOMAG-70k; EDM Millipore, Billerica, MA, United States) as described in previous studies (Lu et al., 2017; Corsi-Zuelli et al., 2019). Previous cytokine studies have shown that prairie vole immune responses are similar to traditional rodent species (Klein and Nelson, 1999; Curtis et al., 2011). Moreover, these studies demonstrate that mouse cytokine antibodies can cross react with prairie vole cytokines and can be measured via ELISA, which is what we used in the current study. The assay was performed at the University of Maryland School of Medicine’s Center for Innovative Biomedical Resources Cytokine Core Laboratory according to manufacturer’s instructions. Cytokines analyzed included Interleukin (IL)-17, IL-4, IL-6, IL-1β, Interferon (IFN)γ, and Vascular Endothelial Growth Factor (VEGF).

### Brain Tissue Preparation

Immediately following cardiac blood sample collection, subjects were transcardially perfused via 0.9% saline followed by 4% paraformaldehyde solution in 0.1M phosphate PB buffer (PB). Their brains were then collected and fixed in 4% paraformaldehyde for 2 h until placed in 30% sucrose in PB at 4°C for storage. The brains were cut into 40 μm coronal sections via sliding microtome.

### Immunocytochemistry

Ionized calcium binding adaptor molecule 1 (Iba-1) is macrophage/microglial-specific molecule involved in the phagocytotic process (Ohsawa et al., 2000). Iba-1 is constitutively expressed in all microglia and is upregulated in active microglia in the brain. The Iba-1 antibody (Wako Chemicals Inc., Richmond, VA) used in this study has been used previously in prairie vole studies (Rebuli et al., 2016; Pohl et al., 2021). Iba-1 staining was performed on sets of coronal sections with 200 μm intervals using previously established methods from our lab (Liu et al., 2019). Briefly, sections were rinsed in 0.1M phosphate-buffered saline (PBS) 3 times for a total 15 min (each rinsing was kept at the rate of 3 times for a total of 15 min below) and then incubated with 1% NaBH_4_ in 0.1 PBS for 10 min. Sections were rinsed and then treated with 0.3% H_2_O_2_ in 0.1M PBS for 20 min. After rinsing in 0.1M PBS, sections were incubated in 10% normal goat serum (NGS, Sigma-Aldrich, St. Luis, MO) in 0.3% Triton X-100 in 0.1M PB (TPBS) for 1 h at room temperature. Subsequently, sections were incubated in rabbit anti-Iba-1 (1:10,000, Wako Chemicals) in 0.3% TPB with 2% NGS at 4°C overnight. Sections were then placed at room temperature for 1 h, rinsed in 0.3% TPBS, and incubated in biotinylated goat antirabbit IgG (1:300, Vector Laboratories, Burlingame, CA) for 2 h at room temperature. Thereafter, sections were rinsed in 0.3% TPBS followed by 0.1M PBS and then incubated in the ABC Elite HRP Kit (Vector Laboratories) in 0.1M PBS for 90 min at room temperature, and Iba-1 immunostaining was revealed using 3′-diaminobenzidine (DAB, Sigma-Aldrich).

### Data Quantification and Analysis

All behaviors were scored via JWatcher software program (V1.0, Macquarie University and UCLA)^1^ and analyzed via two-way ANOVA (SEX x TREATMENT) via GraphPad PRISM. Plasma cytokine levels (pg/ml) were calculated via Luminex Exponent Software. Cytokine data failed tests of normality and were thus analyzed via non-parametric analysis. Means of cytokine levels per animal were analyzed via Kruskal-Wallis tests. Iba-1 labeled cells were quantified in selected brain regions defined by the Paxinos and Watson, 7th edition (Paxinos and Watson, 2014), including the PVN (Plate 23-27), Nucleus Accumbens (NAcc: the core and shell subnuclei; Plate 10-13), Bed Nucleus of the Stria Terminalis (BNST: anterior dorsal and anterior ventral subnuclei; Plate 18-20), Amygdala (AMYG: the cortical (CoA), medial (MeA), and central (CeA) subnuclei; Plate 28-32), Prefrontal Cortex (PFC; Plate 8-11), Dorsal Raphe (DR; Plate 49-53), Lateral Septum (LS; Plate 13-17), and Dentate Gyrus (DG) of the hippocampus: the granular (GrDG), molecular (MoDG), and polymorphic (PoDG) layers; Plate 30-34). These brain regions were chosen due to their demonstrated roles in social behaviors, stress responses, and learning/memory (Sandi and Haller, 2015; Tabbaa et al., 2017; Yavas et al., 2019). The boundary of each nucleus was traced and area was measured by using Stereo Investigator software (MBF Bioscience). Iba-1 positive cells were counted bilaterally from two brain sections per area per subject by using Zeiss Axioskop 2, microscope (Carl Zeiss Microscopy Ltd., Germany), and subsequently converted to cell density (i.e., number of cells per 100 μm^2^ for each brain region) as described previously (Gobrogge et al., 2007; Lieberwirth et al., 2013). The means of Iba-1 density per brain region per animal were analyzed by two-way ANOVA via GraphPad PRISM. Significant sex-by-treatment interactions were further analyzed by the Student-Newman-Keuls *post hoc* tests.

## Results

### Post-weaning Isolation Did Not Affect Anxiety—and Depressive-Like Behavior

**FIGURE 1:**
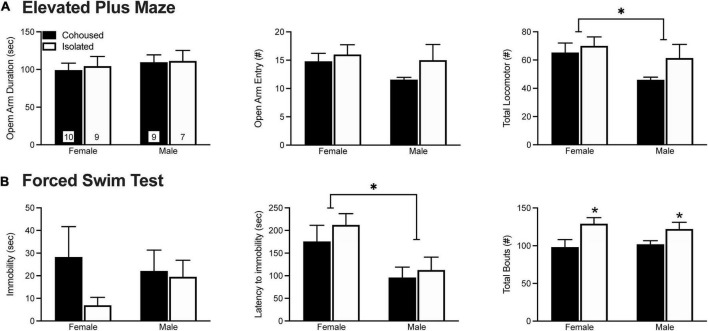
Post-weaning isolation effects on elevated plus maze (EPM) **(A)** and forced swim test (FST) **(B)** behaviors. Six weeks of post-weaning social isolation did not affect the duration on the open arms nor the frequency of open arm entries on the EPM in male and female prairie voles. Females displayed higher levels of overall locomotor activity during the EPM test in comparison to male prairie voles. In the FST, post-weaning social isolation did not affect the duration of immobility in male and female prairie voles. Male prairie voles had significantly lower latency to immobility compared to females, and socially isolated animals had higher overall levels of activity in comparison to cohoused animals. Bars indicate mean ± SEM. The number within each bar on **(A)** indicates the number of animals for each group. * represents *p* < 0.05.

Anxiety-like behavior was assessed in the EPM test (Figure 1A). No treatment effects were found in the duration of time spent in the open arms of the maze [*F*_(1, 31)_ = 0.10, *p* = 0.76] or in the frequency of open arm entries [*F*_(1, 31)_ = 1.98, *p* = 0.17]. A main effect of sex was found, where males showed a lower number of total arm entries in comparison to female voles [*F*_(1, 31)_ = 4.64, *p* < 0.05]. No other main effects of sex, treatment, nor sex-by-treatment interactions were found in any of the EPM measures.

Depressive-like behavior was examined in the FST test (Figure 1B). No treatment effects were found in the duration [*F*_(1, 34)_ = 1.64, *p* = 0.21) or frequency [*F*_(1, 34)_ = 0.98, *p* = 0.33] of immobility. However, isolated voles showed a higher frequency of total bouts in comparison to cohoused voles [*F*_(1, 34)_ = 9.42, *p* < 0.01]. We also found a main effect of sex, where males had a lower latency to immobility in comparison to female voles [*F*_(1, 34)_ = 9.54, *p* < 0.01]. No other significant effects were found in any of the FST measures.

### Post-weaning Isolation Alters Interleukin-1β Plasma Cytokine Levels

**FIGURE 2:**
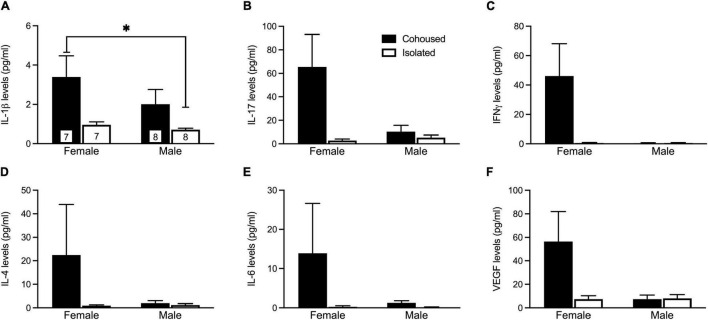
Post-weaning alterations to IL-1β plasma cytokine levels. Isolated males had significantly lower levels of IL-1β **(A)** compared to cohoused females. Although not significant, similar patterns were seen for levels of IL-17 **(B)**, IFNγ **(C)**, IL-4 **(D),** IL-6 **(E),** and VEGF **(F)**. IL-17, Interleukin 17, IFNγ, interferon gamma, VEGF, vascular endothelial growth factor, IL-1β, Interleukin 1 beta, IL-4, interleukin 4, IL-6, interleukin 6. Bars indicate mean ± SEM. The number within each bar on Panel A indicates the number of animals for each group. * represents *p* < 0.05.

The plasma samples were assayed for a variety of cytokine markers previously found to be associated with social isolation (Figure 2). No main effects were found for any cytokines. A significant sex-by-treatment interaction was found for IL-1β (H_3_ = 9.45, *p* < 0.05; Figure 2A), where isolated males had a significantly lower level compared to cohoused females. Further, there was trending interaction significance for IL-17 (H_3_ = 6.72, *p* = 0.08; Figure 2B) and IFNγ (H_3_ = 6.25, *p* = 0.10; Figure 2C). No other sex-by-treatment interactions were found for any measured cytokines (Figure 2).

### Post-weaning Isolation Impacts Microglial Density in a Brain Region-Specific Manner

**FIGURE 3:**
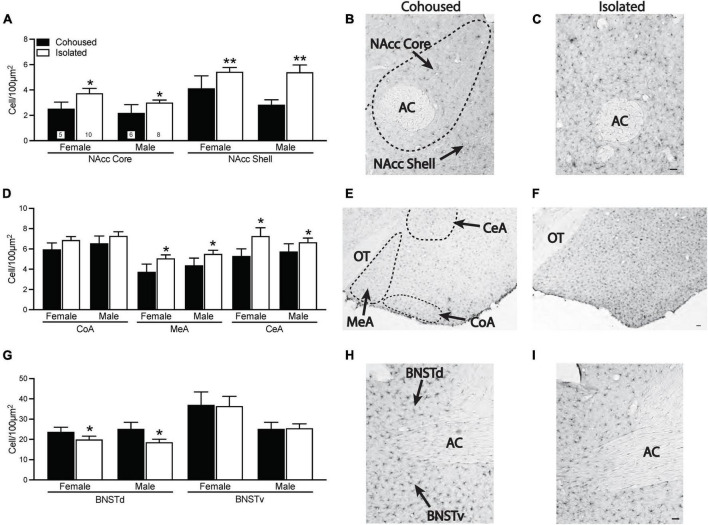
Post-weaning isolation alters ionized calcium binding adaptor molecule 1 (Iba-1) density in a brain region-specific manner. Post-weaning isolation increased Iba-1 cell density in the NAcc Core and Shell **(A–C)** and in the MeA and CeA **(D–F)** of the amygdala. Social isolation also resulted in decreased Iba-1 cell density in the BNSTd, but not BNSTv **(G–I).** Photomicrographs show representative images of Iba-1 staining in the NAcc **(B,C)**, amygdala **(E,F)**, and BNST **(H,I)**. AC, anterior commissure; BNSTd, dorsal region of the bed nucleus stria terminalis; BNSTv, ventral region of the bed nucleus stria terminalis; CeA, central amygdala; CoA, cortical amygdala; MeA, medial amygdala; NAcc, Nucleus Accumbens; OT, optic tract. Bars indicate mean ± SEM. The number within each bar on Panel A indicates the number of animals for each group. * represents *p* < 0.05, ** represents *p* < 0.01. Scale bar = 50 μm.

Iba-1 cell density was quantified from the selected brain regions. Significant treatment effects were found in multiple brain regions (Figure 3). In both the NAcc core [*F*_(1, 25)_ = 5.16, *p* < 0.05] and NAcc shell [*F*_(1, 25)_ = 11.76, *p* < 0.01], isolated voles had significantly higher microglia cell densities compared to cohoused voles (Figures 3A–C). No sex or sex-by-treatment interactions were found in the NAcc core or shell. Isolated voles also had significantly higher density of microglia compared to cohoused voles in both the MeA [*F*_(1, 29)_ = 4.92, *p* < 0.05] and CeA [*F*_(1, 29)_ = 4.34, *p* < 0.05] of the amygdala, but this was not seen in the CoA [*F*_(1, 29)_ = 2.33, *p* = 0.14; Figures 3D–F]. No main effects of sex or sex-by-treatment interactions were found in any measured subnuclei of the amygdala. A main effect of treatment was found in the BNST, where isolated voles showed a significant lower density of microglia in the dorsal BNST [*F*_(1, 31)_ = 5.49, *p* < 0.05], but not the ventral BNST [*F*_(1, 31)_ = 0.42, *p* = 0.52] in comparison to cohoused voles (Figures 3G–I). There were no main effects of sex or sex-by-treatment interactions in the dorsal or ventral BNST.

**FIGURE 4:**
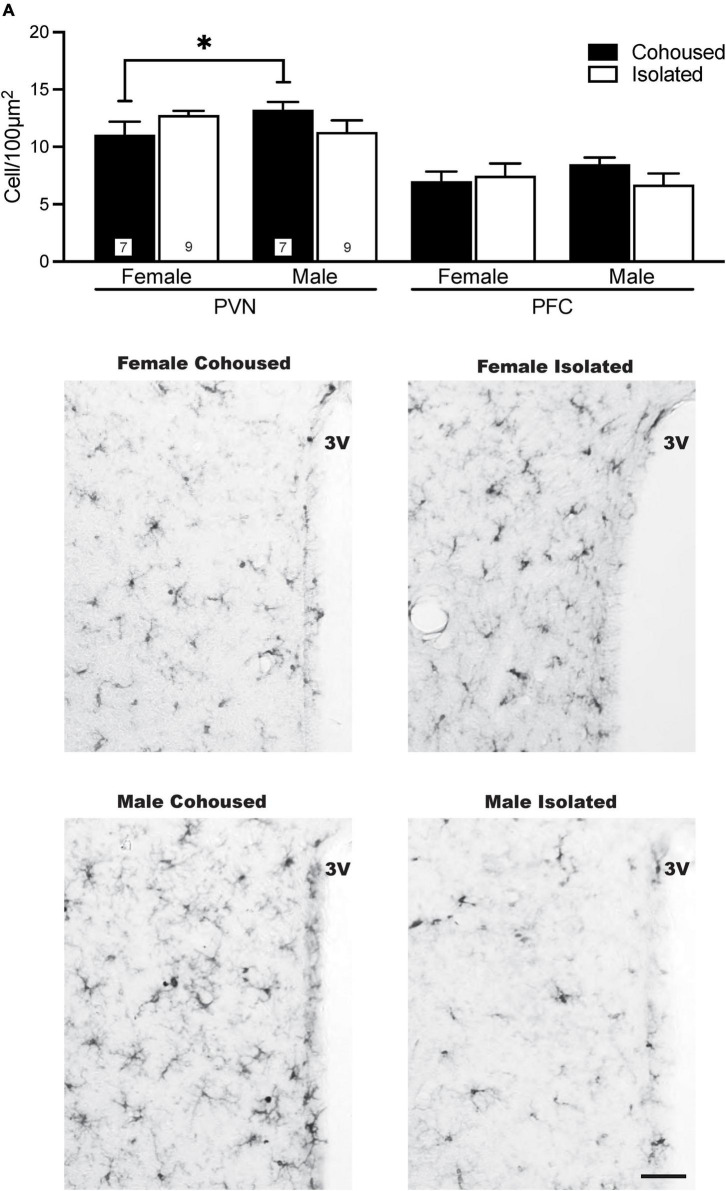
Sex differences in ionized calcium binding adaptor molecule 1 (Iba-1) cell density in the paraventricular nucleus of the hypothalamus (PVN). Cohoused males had higher Iba-1 cell density in the PVN compared to cohoused females, but no differences were found across isolated male or female prairie voles. No group differences in Iba-1 cell density were found in the prefrontal cortex (PFC). Photomicrographs show representative images of Iba-1 labeling in the PVN. 3V, third ventricle. Bars indicate mean ± SEM. The number within each bar on **(A)** indicates the number of animals for each group. * represents *p* < 0.05. Scale bar = 50 μm.

Iba-1 cells were also quantified in the PVN. No significant main effects were present, but a significant sex-by-treatment interaction was found [*F*_(1, 28)_ = 4.61, *p* < 0.01], where cohoused male and female voles differed significantly from each other in the microglial density, but not from isolated males or females (Figure 4). No significant effects of treatment, sex, or sex-by-treatment interactions were found in the PFC (Figure 4) or other quantified brain regions, including the DR, LS, and DG (Table 1).

**TABLE 1:** Iba-1 cell density (cell/100 μm^2^) in various brain regions.

Group	Cohoused		Isolated		*p*		
Sex	M	F	M	F	Group	Sex	*G × S*
DR	17.1 ± 4.6*	10.7 ± 0.7	10.2 ± 1.3	9.8 ± 1.1	ns	ns	ns
LS	6.4 ± 0.8	6.6 ± 1.0	7.3 ± 1.0	8.2 ± 0.6	ns	ns	ns
PoDG	184.9 ± 81.3	193.1 ± 59.2	234.5 ± 57.3	202.6 ± 47.7	ns	ns	ns
MoDG	130.8 ± 28.9	193.2 ± 56.8	235.8 ± 51.9	173.6 ± 32.4	ns	ns	ns
GrDG	81.8 ± 25.2	121.9 ± 36.0	131.7 ± 40.8	95.4 ± 16.6	ns	ns	ns

Iba-1, ionized calcium-binding adapter molecule 1; DR, dorsal raphe; LS, lateral septum; PoDG, polymorphic layer of the dentate gyrus; MoDG, molecular layer of the dentate gyrus; GrDG, granular layer of the dentate gyrus. *Mean ± SEM.

## Discussion

Post-weaning social isolation, a chronic stressor during development, has been shown to induce behavioral and physiological stress responses—especially in social animals (Fone and Porkess, 2008; Matsumoto et al., 2019; Walker et al., 2019). In the present study, post-weaning isolation did not significantly alter anxiety-like or depressive-like behaviors compared to cohoused male and female prairie voles. However, isolated male voles showed a lower level of IL-1β in compared to cohoused females. Isolated voles also showed higher microglial densities in the NAcc, MeA, CeA, but a lower microglial density in the dorsal BNST compared to cohoused voles. These results suggest that chronic social isolation during a juvenile window may have distinctive effects on both peripheral and central immune function between male and female prairie voles.

Previous findings have shown that isolation increases anxiety-like and depressive-like behaviors in prairie voles (Grippo et al., 2008; Pan et al., 2009) as well as in traditional rodent models (Amiri et al., 2015; Huang et al., 2017). Therefore, we were surprised to see no significant changes in anxiety-like and depressive-like behaviors by post-weaning social isolation in the present study. We don’t have ready explanations for such discrepancies and can only speculate that these differences in results may have been due to subtle differences in the experimental designs and procedures. The type, number, timing, and order of behavioral tests conducted in previous studies were different from our current study, which may contribute to these differences. For example, in a previous study (Pan et al., 2009), male prairie voles underwent the social affiliation, open field, and EPM tests (over three consecutive days) after 6 weeks of post-weaning isolation. Moreover, the majority of social isolation studies in prairie voles have been done in adult voles, which may contribute to the difference in results. In addition, our vole breeding colony has recently been outbred with wild-captured prairie voles. This outbreeding process is essential for maintaining genetic variation across prairie vole laboratories. Whether such outbreeding has generated colony-specific behavioral responses to social isolation needs to be further examined. Nevertheless, post-weaning isolation increased total bouts in the FST in both male and female voles in the present study. Although much of the social isolation literature show that social isolation increases immobility in the FST (Lukkes et al., 2009b; Kokare et al., 2010; Sargin et al., 2016), there are numerous studies that demonstrate hyperactivity after post-weaning isolation (Fone and Porkess, 2008; Ashby et al., 2010; Hickey et al., 2012). It should be noted that we chose to administer the FST second, as it is more stress-inducing than the EPM test. However, we cannot exclude the possibly that the EPM test might still have residual effects 24 h later, or that post-weaning isolation also may have resulted in dysregulated behaviors not illustrated by the EPM and FST tests.

To our knowledge, the present study is the first study to examine peripheral cytokines post-isolation in male and female prairie voles. In mice, cytokine expression peaks 6–12 h after one acute stressor, and 1 h after two acute stressors spaced 24 h apart (similar to our current study paradigm) (Cheng et al., 2015). As plasma cytokines were collected immediately after the 5-min FST, our data should be better explained by the effects of social housing conditions rather than from the acute FST stressor. Our data indicate a sex by treatment interaction, where isolated male voles had lower levels of IL-1β compared to cohoused females. This is surprising, given that IL-1β is a key cytokine in the induction of the sickness behavioral responses, which includes HPA axis activation, fever, and social withdrawal (Dantzer, 2001, 2006). However, a previous post-weaning isolation experiment in rats found that contrary to the expected proinflammatory phenotype seen in socially isolated adults, the measured cytokines (e.g., IL-10, IL-6, TNF-α) either stayed the same or decreased (Corsi-Zuelli et al., 2019), supporting our data that post-weaning isolation can result in lower levels of peripheral cytokines. Perhaps this reduction in IL-1β also plays a role in the lack of significant differences in behavior through compensatory mechanisms resulting from the stressful isolation experience. IL-1β’s relationship with the stress response, and more specifically corticosterone (CORT), is also worth noting. Previous prairie vole studies using the 6-week isolation paradigm have found elevated CORT levels from the isolation experience in adult, male prairie voles (Donovan et al., 2020), but not in adult females (Lieberwirth et al., 2012; Donovan et al., 2020). Intriguingly, it has been shown that pretreating animals with varying levels of CORT changes the levels of IL-1β released in response to an immune challenge—emphasizing their dynamic relationship (Liu et al., 2018). The high level of IL-1β seen in cohoused females in the present study may be due to the fact that prairie voles, and particularly female voles, naturally have much higher levels of circulating CORT than other traditional laboratory rodents (Taymans et al., 1997). Further, IL-1β is expressed at high levels in the brain during development (Bilbo and Schwarz, 2009) and is essential for normal cognitive function (Giulian et al., 1988; Bilbo and Schwarz, 2012). IL-1β is responsible for many downstream immune targets and effects, one of which is the production of IL-17 (Kuwabara et al., 2017; Pyrillou et al., 2020). Likewise, IL-17 can also induce production of IL-1β (Xu and Cao, 2010). Therefore, this bidirectional feedback between cytokines in their release and production may be an important factor when considering the functional importance of peripheral cytokines in shaping central function and resulting behaviors.

Although only trending toward significance, our additional data show a similar pattern, where female voles isolated after weaning tend to have lower plasma levels of IFNγ and IL-17 compared to cohoused females. The lack of significance in these results may be due to either small sample sizes or large individual variation, which is common in prairie voles (Hammock et al., 2005; Barrett et al., 2013; Donovan et al., 2020). Although high levels of plasma cytokines may suggest a proinflammatory state, metadata analysis of transcriptomes found that group-housed rats and mice have upregulated IFNγ gene signature, and social isolation greatly reduces IFNγ (Krügel et al., 2014). Moreover, IFNγ- and IFNγ receptor-deficient mice display social deficits, and IFNγ signaling controls neural circuits shaping social behavior (Filiano et al., 2016). Further, IL-17 has been dubbed as the “social cytokine” (Hoogenraad and Riol-Blanco, 2020) due to its role in rescuing social deficits in mouse models of neurodevelopmental disorders (Reed et al., 2020). Given the critical role the immune system plays in shaping neuronal circuits during development (Morimoto and Nakajima, 2019), high levels of IFNγ and IL-17 in cohoused females may be shaping typical behaviors, whereas a reduction in these peripheral immune markers may point to the potentially damaging effect of social isolation on neuronal health and development during a critical period.

As social isolation has been shown to alter immune function in adult prairie voles (Scotti et al., 2015) and is also associated with sex- and brain region-specific alterations in microgliosis (Donovan et al., 2020), we were interested in seeing how post-weaning isolation altered microglial density in key brain regions known to be affected by isolation stress. We found significant differences in microglial density in a brain region-specific pattern, where isolated voles had significantly higher levels of microglial densities in the NAcc, MeA, and CeA, but lower microglial density in the dorsal BNST compared to cohoused voles. These data add to the growing literature that isolation during adolescent timeframes can alter central immune function in various rodent species (Wang H. T. et al., 2017; Dunphy-Doherty et al., 2018; Gong et al., 2018).

The NAcc has been linked to microglial-specific alterations after various stressors, with both chronic restraint stress and social isolation resulting in increased levels of microglial activation in this brain region (Tynan et al., 2010; Donovan et al., 2020). Moreover, it has been shown that neurochemical systems in the NAcc, such as CRF and OT, are critical for partner loss in prairie voles (Bosch et al., 2016), and microglia can also directly interact with reward systems critical for pair bonding (Loth and Donaldson, 2021). As the NAcc has been shown to be an important node for post-weaning-induced alterations (Bendersky et al., 2021), we speculate that changes in microglia in the NAcc play a role in alterations to neuronal circuits and behaviors. Further, it has been reported that post-weaning isolation reduces the size of neurons and volume of the MeA (Cooke et al., 2000), and microglia play a causal role in modulating underlying neurochemical alterations and behavioral outcomes from isolation stress (Wang H. T. et al., 2017; Gong et al., 2018). Given that our data show an isolation-induced increase in microglial density in the MeA, it would be interesting to assess whether the increased presence of microglia plays a causal role in amygdala neuronal alterations and their underlying behaviors. Finally, although there are little data focusing on microglial alterations after isolation in the BNST, one study found that aged rats have significantly higher density of microglia in the BNST, indicating that BNST microglial alterations may play a role in shaping age-related circuits in the brain (Perkins et al., 2018). A study in mice also showed that acute social isolation can blunt long-term potentiation in the dorsal-lateral BNST (Conrad et al., 2011). Thus, our data showing lower microglial density in the dorsal BNST after isolation during development support the idea that the BNST may serve as a critical node in the underlying circuitry driving age- and isolation-induced changes to behavior.

Another interesting finding in our study is the notable sex differences in behavior as well as in both peripheral and central immune measures. We saw sex differences in both behavioral assays, where females displayed higher levels of locomotor activity on the EPM and a longer latency to immobility in the FST. Sex differences in activity levels after stress have also been found in previous studies, suggesting that males and females may have differential outcomes with hyperactivity (West and Michael, 1988; Yamaura et al., 2013). We have also found a sex by treatment interaction in IL-1β cytokine levels. These differences seen in levels of IL-1β were particularly fascinating given previously established sex differences in inflammation and immunity (Ahnstedt and McCullough, 2019). These sex differences in our data could reflect differences in gonadal steroids between males and females, given that gonadal steroids can promote the expression of immune genes and can thus mediate the immune response (Klein and Flanagan, 2016; Ahnstedt and McCullough, 2019). Interestingly, a previous study found that developmental exposure to synthetic estrogen can alter sex-specific colonization by microglia in a brain region-specific manner in prairie voles (Rebuli et al., 2016). Further, in the present study, we found that control males have higher microglial density in the PVN compared to control females, which is consistent with our finding in adult prairie voles (Donovan et al., 2020). A recent study demonstrated that parvocellular PVN microglial priming occurs in male, but not female, prairie voles after partner loss (Pohl et al., 2021). In addition, neurochemical and gene expression in the PVN is altered by social isolation during adolescence in male voles (Pan et al., 2009). Together, these data support the notion that the PVN may be particularly important for driving sexually dimorphic, immune-related mechanisms in the brain.

In summary, our data, for the first time, demonstrate that 6 weeks of social isolation immediately after weaning is associated with central and peripheral immune alterations in prairie voles in a sex-, brain region-, and cytokine-specific manner. These data add to the growing literature suggesting that social isolation can have long-lasting impacts on central and peripheral immune function, and that these, along with other system-wide effects (Grippo et al., 2007b; McNeal et al., 2014), may be ultimately leading to negative health outcomes. As previous research demonstrate a casual role of microglia shaping sex-specific social behaviors during development (Kopec et al., 2018), more research must be done to explore how microglia and/or peripheral cytokines may modulate sex differences in male and female prairie vole social behavior as well.

## Data Availability Statement

The raw data supporting the conclusions of this article will be made available by the authors, without undue reservation.

## Ethics Statement

The animal study was reviewed and approved by the Institutional Animal Care and Use Committee at Florida State University.

## Author Contributions

MD and ZW designed the study. MD, EC, and YL conducted the experiments. MD and ZW analyzed the data and wrote the manuscript. All authors read and approved the manuscript.

## Conflict of Interest

The authors declare that the research was conducted in the absence of any commercial or financial relationships that could be construed as a potential conflict of interest.

## Publisher’s Note

All claims expressed in this article are solely those of the authors and do not necessarily represent those of their affiliated organizations, or those of the publisher, the editors and the reviewers. Any product that may be evaluated in this article, or claim that may be made by its manufacturer, is not guaranteed or endorsed by the publisher.

## Footnotes

1
http://www.jwatcher.ucla.edu/
